# Neurogenic and pericytic plasticity of conditionally immortalized cells derived from renal erythropoietin‐producing cells

**DOI:** 10.1002/jcp.30677

**Published:** 2022-01-10

**Authors:** Andreas M. Bapst, Thomas Knöpfel, Karen A. Nolan, Faik Imeri, Claus D. Schuh, Andrew M. Hall, Jia Guo, Dörthe M. Katschinski, Roland H. Wenger

**Affiliations:** ^1^ Institute of Physiology University of Zürich Zürich Switzerland; ^2^ National Centre of Competence in Research “Kidney.CH” University of Zürich Zürich Switzerland; ^3^ Institute of Anatomy University of Zürich Zürich Switzerland; ^4^ Institute for Cardiovascular Physiology, University Medical Center Göttingen Georg‐August‐University Göttingen Germany; ^5^ Present address: Faik Imeri, Department of Nephrology and Hypertension Bern University Hospital Bern Switzerland

**Keywords:** erythropoietin, hypoxia, kidney, oxygen sensing, stem cell

## Abstract

In adult mammals, the kidney is the main source of circulating erythropoietin (Epo), the master regulator of erythropoiesis. In vivo data in mice demonstrated multiple subtypes of interstitial renal Epo‐producing (REP) cells. To analyze the differentiation plasticity of fibroblastoid REP cells, we used a transgenic REP cell reporter mouse model to generate conditionally immortalized REP‐derived (REPD) cell lines. Under nonpermissive conditions, REPD cells ceased from proliferation and acquired a stem cell‐like state, with strongly enhanced hypoxia‐inducible factor 2 (HIF‐2α), stem cell antigen 1 (SCA‐1), and CD133 expression, but also enhanced alpha‐smooth muscle actin (αSMA) expression, indicating myofibroblastic signaling. These cells maintained the “on‐off” nature of Epo expression observed in REP cells in vivo, whereas other HIF target genes showed a more permanent regulation. Like REP cells in vivo, REPD cells cultured in vitro generated long tunneling nanotubes (TNTs) that aligned with endothelial vascular structures, were densely packed with mitochondria and became more numerous under hypoxic conditions. Although inhibition of mitochondrial oxygen consumption blunted HIF signaling, removal of the TNTs did not affect or even enhance the expression of HIF target genes. Apart from pericytes, REPD cells readily differentiated into neuroglia but not adipogenic, chondrogenic, or osteogenic lineages, consistent with a neuronal origin of at least a subpopulation of REP cells. In summary, these results suggest an unprecedented combination of differentiation features of this unique cell type.

## Graphical abstract

Conditionally immortalized REP cell‐derived (REPD) cell lines derived from renal Epo‐producing (REP) cells ceased from proliferation and acquired a stem cell‐like state under nonpermissive conditions, with enhanced hypoxia‐inducible factor 2 (HIF‐2α), stem cell antigen 1 (SCA‐1), and CD133 expression. Like REP cells in vivo, REPD cells cultured in vitro generated long nanotubes that aligned with endothelial vascular structures, were densely packed with mitochondria, and became more numerous under hypoxic conditions. REPD cells readily differentiated into neuroglia but not mesenchymal lineages, confirming the neuron‐like features of REP cells.

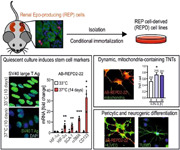

## 1 INTRODUCTION

The erythropoietic hormone erythropoietin (Epo) is essential for red blood cell (RBC) homeostasis (Wenger & Kurtz, 2011). The main source of Epo in adult mammals are renal Epo‐producing (REP) cells located in the peritubular interstitial space of the corticomedullary border region (Nolan & Wenger, 2018). Epo is primarily regulated on the messenger RNA (mRNA) level, mediated by the transcription factor hypoxia‐inducible factor 2 (HIF‐2) (Kapitsinou et al., 2010; Scortegagna et al., 2005). Upon a reduction in blood oxygen concentration, REP cells transiently induce Epo production, which increases blood Epo levels up to several hundredfolds (Ebert & Bunn, 1999).

Intriguingly, renal Epo production and blood Epo levels diminish rapidly despite ongoing hypoxia, long before a corrective response in blood oxygen transport capacity occurs (Dahl et al., 2022; Eckardt et al., 1990). Transcriptional Epo activity in REP cells seems to follow a poorly understood “on‐off” pattern (Souma et al., 2015; Suzuki & Yamamoto, 2016). The temporal mismatch between the “on” state and tissue hypoxia suggests a REP cell‐intrinsic negative feedback regulation unrelated to RBC counts, presumably mediated by hypoxia‐inducible prolyl hydroxylase domain (PHD) enzymes (Wenger & Hoogewijs, 2010). We and others have previously provided evidence for the existence of such an intrinsic negative feedback loop in cancer cells in vitro (Marxsen et al., 2004; Stiehl et al., 2006). Despite ongoing hypoxia, an early PHD3 and a later PHD2 induction functionally limited HIF activity in these cells (Stiehl et al., 2006). A similar mechanism has also been suggested for PHD3 in vivo using erythrocytotic PHD2/3 knock‐out mice and in vitro using mouse embryonic fibroblasts (MEFs) (Minamishima et al., 2009). Not only the increase in the PHD levels, as evidenced by small interfering RNA (siRNA)‐mediated PHD1/2/3 knock‐down in vitro and in vivo, but also in intracellular oxygen availability has been suggested to desensitize HIF under chronic hypoxia (Ginouvès et al., 2008). However, no kinetics experiments under hypoxic conditions to analyze the negative feedback loop in HIF signaling have been performed in these studies. As this is difficult to achieve in the rare REP cells in vivo, it would be of interest to analyze the expression kinetics and role of PHD2/3 in REP cell‐derived (REPD) cell lines upon exposure to hypoxia.

During end‐stage renal disease (ESRD) Epo production by REP cells is impaired, resulting in renal anemia (Koury & Haase, 2015). It is not clear why REP cells cease producing Epo during ESRD. Impaired kidney function during ESRD could result in local tissue hyperoxia due to severely reduced oxygen consumption, preventing an appropriate Epo response (Farsijani et al., 2016). EPO expression could also be lost due to paracrine signals and transdifferentiation of REP cells, as indicated by the increased expression of the myofibroblast marker alpha‐smooth muscle actin (αSMA) during a model of ESRD in transgenically modified reporter mice (Souma et al., 2013). Indeed, a REPD cell line derived from the same mouse model lost platelet‐derived growth factor receptor β (PDGFRβ) and gained αSMA, suggesting that transdifferentiation was the cause for the parallel loss of HIF‐2 and Epo expression (Sato,Hirano, et al., 2019).

To elucidate the mechanisms underlying negative feedback regulation of the PHD‐HIF cascade, and to analyze the cellular differentiation plasticity, an appropriate non‐proliferative REPD cell culture model would be invaluable. Although liver‐ and brain‐derived cancer cell lines with permanent hypoxia‐inducible Epo expression are well‐established (Orlando et al., 2020), according to our and others' experience primary REP cells and nonconditionally immortalized REPD cell lines tend to lose hypoxia‐inducible Epo production during in vitro cultivation (Bussolati et al., 2013; Imeri et al., 2019; Maxwell et al., 1993; Olmos et al., 2018; Sato, Hirano, et al., 2019), consistent with the “on‐off” behavior of REP cells in vivo (Souma et al., 2013, 2015; Suzuki & Yamamoto, 2016). Regarding the spatial residency, stochastic recruitment, and transient nature of Epo expression in vivo, loss of Epo production in in vitro cultured REPD cells probably has to be expected, limiting some—but not all—mechanistic insights that can be obtained with these cell lines.

We recently established three independent cell lines derived from busy (i.e., with active *Epo* gene loci) REP cells, assuming that under these conditions a more permanent Epo expression could have been expected. Indeed, these fibroblastoid atypical interstitial kidney (FAIK) cell lines recapitulated the telocyte morphology and marker profile of REP cells, including markers associated with fibroblasts, pericytes, neurons, and hematopoietic progenitors (Imeri et al., 2019). Like REP cells in vivo, FAIK cells demonstrated rapid HIF‐2α stabilization kinetics as well as hypoxia‐inducible, transient Epo protein regulation. However, the vast majority of these nonconditionally immortalized and rapidly proliferating FAIK cells turned into “off” cells and Epo production declined during permanent cell culture.

As REP cells in vivo normally do not proliferate (Dahl et al., 2022; Souma et al., 2013), affecting oxygen consumption and energy metabolism, it is possible that cellular quiescence is required for maintaining normal oxygen sensing and signaling in cultured REPD cells. Similar observations have been made before in other cell lines, for example, derived from podocytes or kidney proximal tubule cells: conditional immortalization using a heat‐labile SV40 large T antigen followed by cultivation under nonpermissive conditions (i.e., an increase in the temperature from 33°C to 37–39°C) resulted in a differentiation pattern more closely resembling the respective cell type in vivo (Loghman‐Adham et al., 1997; Saleem et al., 2002). Therefore, we recently generated conditionally immortalized REPD cell lines (Bapst et al., 2020). Here, we investigated the in vitro differentiation of these cells under nonpermissive and lineage supporting cell culture conditions, and we analyzed the role of mitochondria and tunneling nanotubes (TNTs) in oxygen sensing and signaling.

## 2 MATERIALS AND METHODS

### 2.1 Animals

The generation of *Epo‐Cre* ^ERT2^ *#1* ^tg/tg^x*Rosa26* ^tdT/Terminator^ mice has been described previously (Imeri et al., 2019). For Cre activation, 200 mg/kg tamoxifen (Sigma‐Aldrich) was administered by daily gavage for 5 days, followed by exposure to 0.1% inspiratory carbon monoxide (CO) in air for 4 h. *Epo‐Cre* ^ERT2^ *#241* ^tg/tg^x*Rosa26* ^tdT/tdT^ mice were used for intravital imaging 13 weeks after Cre activation. All animal experiments were approved by the veterinary office of the Canton Zürich (license numbers ZH233/15 and ZH085/2019).

### 2.2 Intravital imaging

The left kidney was externalized and imaged by intravital microscopy as published previously (Schuh et al., 2016). To label perfused blood vessels, serum transferrin (which is not filtered by the kidneys) coupled to Alexa 647 (T23366; Thermo Fisher Scientific) was injected into the internal jugular vein. Imaging was performed using a custom‐built multiphoton microscope operating in an inverted mode (Mayrhofer et al., 2015).

### 2.3 Tissue processing and imaging

Mice were killed by cervical dislocation and the kidneys were fixed with 4% paraformaldehyde. Cryosections (12–20 μm) and cultured cells were analyzed by immunofluorescence as described previously (Bapst et al., 2020; Imeri et al., 2019), using the antibodies listed in Table S1. mRNA fluorescence in situ hybridization (mRNA‐FISH) was performed as described previously (Dahl et al., 2022).

### 2.4 Cell culture

The production of REPD cell lines has been described in detail previously (Bapst et al., 2020; Imeri et al., 2019). For hypoxic exposure (0.2% O_2_ and 5% CO_2_ in humidified N_2_) cells were incubated in an InvivO2 400 workstation (Baker Ruskinn). For mitochondria analyses, AB‐REPD2‐22 were stably transfected with pLYS1‐FLAG‐mitoGFP‐HA (kind gift from Vamsi Mootha; plasmid #50057; Addgene). For TNT quantification, AB‐REPD2‐22mitoGFP cells were cultivated under nonpermissive (37°C) conditions for 14 days before exposure to normoxia or hypoxia (0.2% O_2_). Cell morphology was analyzed using endogenous tdTomato fluorescence, and the number and length of the TNTs (minimal length 25 μm; maximal diameter 2 μm) was assessed. For coculture experiments, human umbilical vein endothelial cells (HUVECs) (Lonza) were cultured in endothelial cell growth basal medium‐2 (EBM 2) supplemented with endothelial growth media‐2 (EGM 2) Bullet kit (CC‐4176+CC‐3156; Lonza) and stably transfected with pLenti6.3/V5‐DEST‐GFP (kind gift from Lynda Chin; plasmid #40125; Addgene). HUVEC green fluorescent protein (HUVEC‐GFP) cells were coplated with AB‐REPD2‐22 cells (10:1) on solidified matrigel (354234; Corning). For differentiation analyses, REPD cells were cultured under nonpermissive (37°C) conditions for 7 days, before cultivation in the respective differentiation medium (all from Promocell) for adipogenesis (C‐28016; 21 days), chondrogenesis (C‐28012; 21 days), osteogenesis (C‐28013; 14 days), neurogenesis (C‐28015; 3 days), or kept in mesenchymal stem cell (MSC) growth medium 2 (C‐28009). Cells were stained with sudan III, alizarin red S, or alcian blue as described by the manufacturer (Sigma‐Aldrich). MSCs derived from adipose tissue served as differentiation controls (PT‐5006; Lonza).

### 2.5 RNA and protein analyses

RNA was extracted and quantified by reverse‐transcription (RT) real‐time quantitative (q) PCR as described previously (Imeri et al., 2019). Transcript levels were normalized to mouse ribosomal protein L28 or β‐actin mRNA. Primers used for RT‐qPCR are listed in Table S2 and were purchased from Microsynth. Immunoblot detection of HIFα proteins has been performed as reported previously (Müller‐Edenborn et al., 2015), using the antibodies listed in Table S1.

### 2.6 Focused ion beam scanning electron tomography (FIB‐SEM)

FIB‐SEM was essentially performed as previously described (Zumthor et al., 2016), except that cells were primarily fixed with 2.5% glutaraldehyde.

## 3 RESULTS

### 3.1 Increased HIF‐2α and stem cell markers in conditionally immortalized REPD cells under nonpermissive conditions

**Figure 1:**
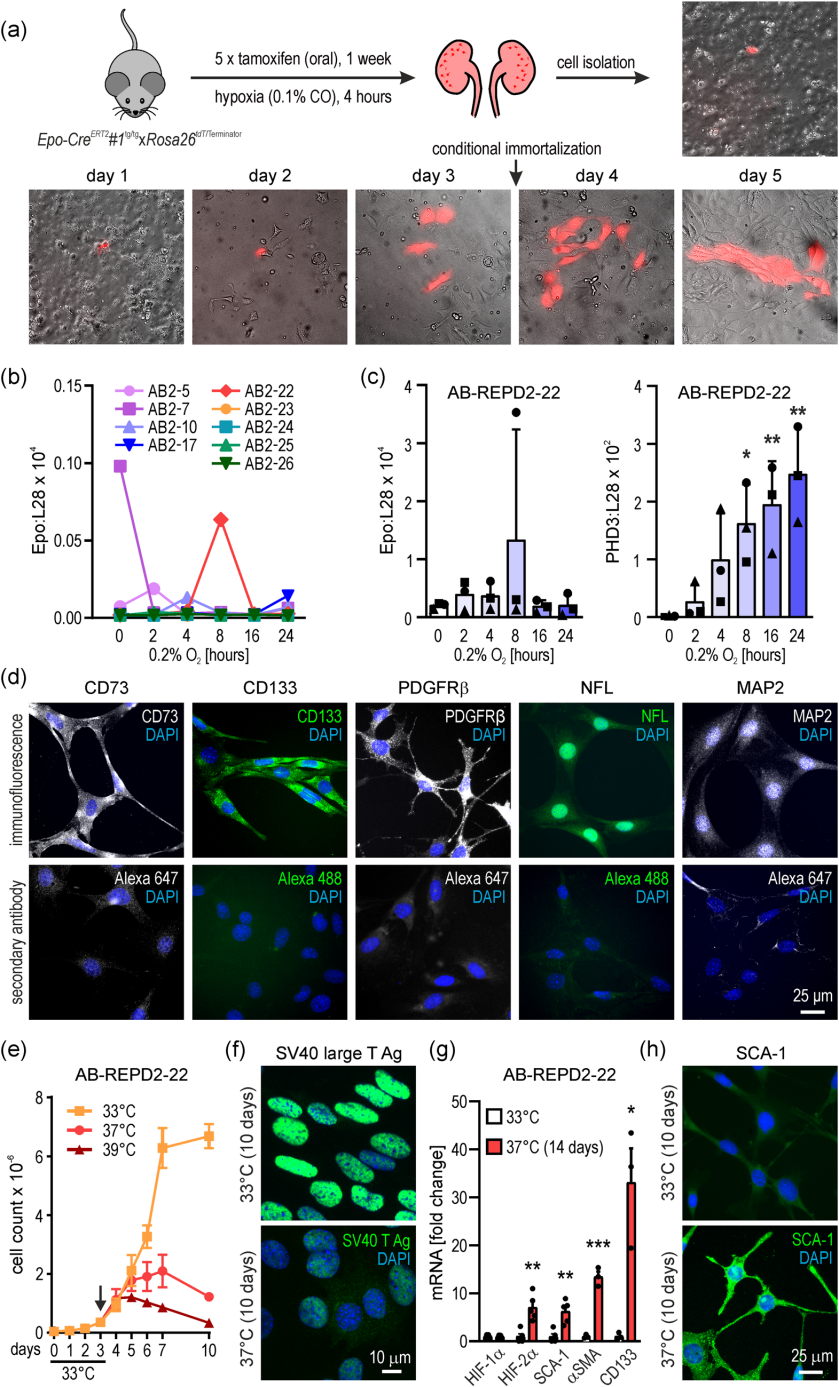
Generation and characterization of conditionally immortalized REPD cell lines. (a) Schematic indicating the generation of AB‐REPD cells. Four days after the in vivo carbon monoxide (CO) stimulus, in vitro proliferating primary tdTomato+ cells (red) were immortalized. (b) A subset of nine clonal cells derived from the conditionally immortalized AB‐REPD2 cell pool was cultivated under nonpermissive (39°C) conditions for 3 days and analyzed for hypoxic Epo mRNA induction by RT‐qPCR. Data are shown relative to the ribosomal protein L28 mRNA levels. (c) "On‐off" behavior of AB‐REPD2‐22 cells. Epo and PHD3 mRNA levels were determined as in (b); shown are mean values + *SD* of *n* = 3 independent experimental series which are indicated by common symbols. One‐way ANOVA with Dunnett's multiple comparisons test was used to evaluate hypoxic induction (*, *p* < 0.05; **, *p* < 0.01). (d) Indirect immunofluorescence (upper panel) or secondary antibody controls (lower panel) of the indicated marker proteins in AB‐REPD2‐22 cells cultivated for 3 days under nonpermissive (39°C) conditions. Nuclei were stained by DAPI (blue). (e) Proliferation of AB‐REPD2‐22 cells cultivated under permissive (33°C, *n* = 3) conditions or switched to nonpermissive (37°C, *n* = 3; or 39°C, *n* = 1) conditions as indicated (arrow). Shown are mean values ± *SD*. (f) Nuclear localization of the heat‐labile SV40 large T antigen under permissive (33°C) or nonpermissive (37°C) conditions. (g) Induction of the indicated mRNAs by exposure to nonpermissive (37°C) conditions for 14 days. Transcript levels were determined by RT‐qPCR and normalized to β‐actin levels. Shown are mean induction factors + SD of *n* = 3−5 experiments. Student unpaired *t* tests were used to statistically evaluate differences to the permissive (33°C) conditions (*, *p* < 0.05; **, *p* < 0.01; ***, *p* < 0.001). (h) SCA‐1 immunofluorescence under permissive (33°C) or nonpermissive (37°C) conditions. ANOVA, analysis of variance; DAPI, 4', 6‐diamidino‐2‐phenylindole; CD73, ecto‐5'‐nucleotidase; CD133, prominin‐1; Epo, erythropoietin; PDGFRβ, platelet‐derived growth factor receptor β; NFL, neurofilament light polypeptide; MAP2, microtubule‐associated protein 2; PHD3, prolyl hydroxylase domain 3; HIF, hypoxia‐inducible factor; SCA‐1, stem cell antigen 1; αSMA, α smooth muscle actin; REPD, REP cell‐derived; RT‐qPCR, reverse transcription quantitative polymerase chain reaction

Using a previously established protocol (Bapst et al., 2020; Imeri et al., 2019), tdTomato (tdT)‐positive REP cells were isolated from *Epo‐Cre* ^ERT2^ *#1* ^tg/tg^x*Rosa26* ^tdT/Terminator^ mice and immortalized with a heat‐labile SV40 large T antigen to generate REPD cell lines (Figure 1a). Of 52 clonal cell lines, 9 have been analyzed for hypoxic Epo expression after cultivation under nonpermissive conditions (39°C) for 3 days (Figure 1b). However, Epo mRNA was only sporadically found to be induced in these clonal cell lines, and originally “on” AB‐REPD2‐22 cells repeatedly turned into the “off” state upon replication of the exposure to hypoxia (Figure 1c), whereas the expression of a hypoxia‐inducible control gene (PHD3) (Figure 1c), as well as established REP protein markers (CD73, CD133, PDGFRβ, NFL, MAP2), was maintained (Figure 1d). Upon switching from permissive (33°C) to nonpermissive (37°C or 39°C) conditions, AB‐REPD2‐22 cells stopped to proliferate (Figure 1e) and the SV40 large T antigen disappeared from the nuclei (Figure 1f). Because 37°C sufficiently inhibited the large T antigen, this temperature was used for all further experiments, avoiding heat stress. AB‐REPD2‐22 cells displayed a marked upregulation of HIF‐2α but not HIF‐1α mRNA, and they acquired a stem cell‐like state, with strongly enhanced stem cell antigen 1 (SCA‐1) (Ly‐6A/E) and CD133 (prominin‐1) induction (Figure 1g). Intriguingly, while the MSC‐like markers CD29, CD81, and basigin largely remained unaffected (data not shown), mRNA levels of the myofibroblast marker α smooth muscle actin (αSMA) (Acta2) were also increased under nonpermissive conditions. Immunofluorescence microscopy confirmed the increase of the SCA‐1 stem cell marker under nonpermissive (37°C, 10 days) conditions (Figure 1h). Overlapping SCA‐1 and Epo mRNA expression in REP cells was also confirmed in vivo (Figure S1).

### 3.2 Oxygen sensing and HIF signaling in conditionally immortalized REPD cells

**Figure 2:**
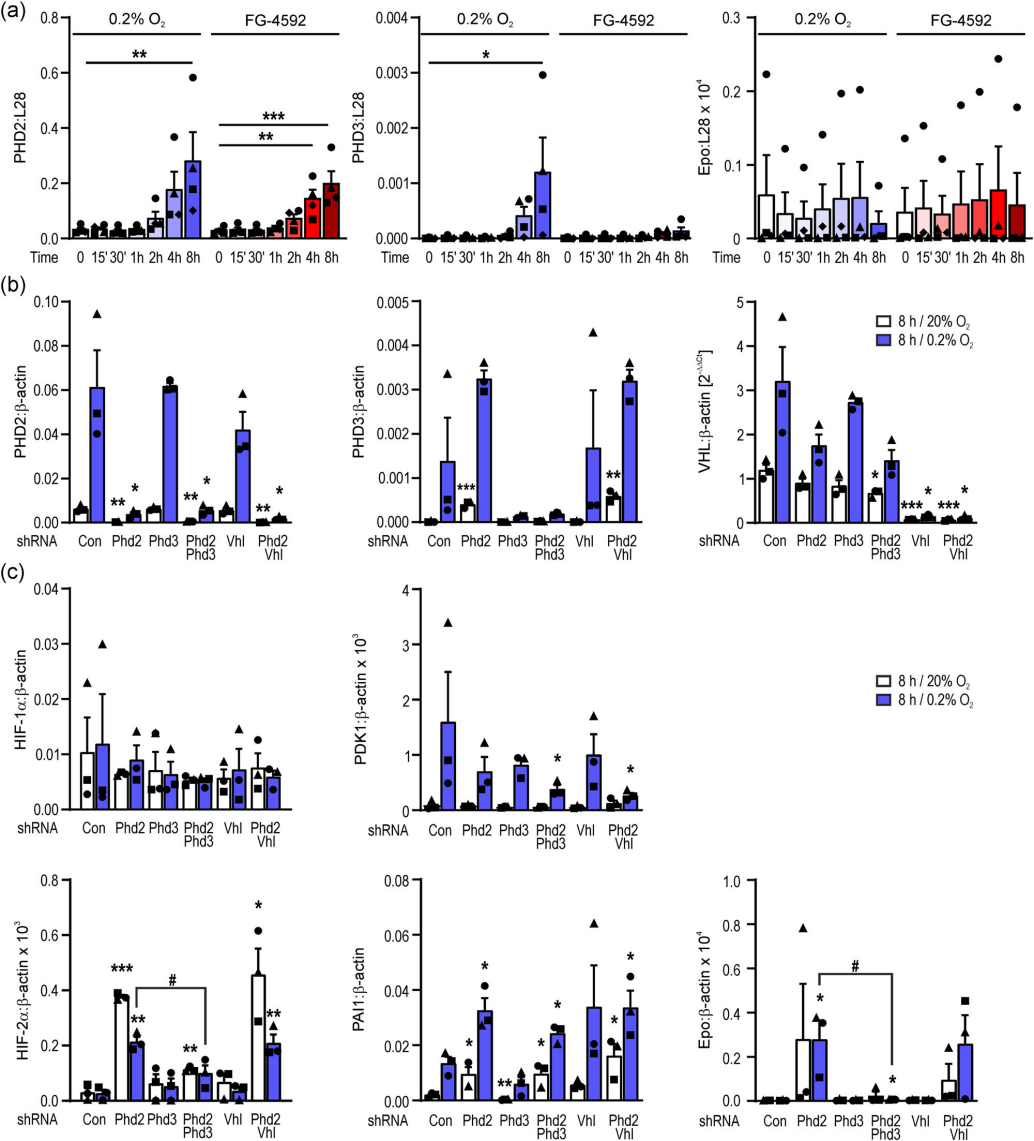
Oxygen signaling in REPD cells. (a) AB‐REPD2‐22 cells were cultured for 14 days under nonpermissive (37°C) conditions and exposed to hypoxia or FG‐4592 as indicated. mRNA levels were determined by RT‐qPCR and are displayed relative to the ribosomal protein L28 mRNA levels (mean + SEM, *n* = 4 independent experimental series which are indicated by common symbols). Two‐way ANOVA with Dunnett's multiple comparisons test was used to evaluate hypoxic induction (*, *p* < 0.05; **, *p* < 0.01; ***, *p* < 0.001). (b) Silencing of the PHD oxygen sensors and VHL in AB‐REPD2‐22 cells by stable transfection with the indicated shRNA constructs. Following cultivation under nonpermissive (37°C) conditions for 14 days, cells were exposed to hypoxia and mRNA levels were determined by RT‐qPCR. (c) mRNA levels of HIF‐1α and the HIF‐1 target PDK1 (upper panel), and HIF‐2α and the HIF‐2 targets PAI1 and Epo (lower panel) in the indicated knock‐down cells. (b, c) Transcript levels are shown relative to β‐actin mRNA (mean + SEM, n = 3). The delta‐delta Ct method ($2^{‐\Delta \Delta C_{t}}$) was used where no internal standard was available. Student unpaired *t* tests were used to statistically evaluate differences to the respective control shRNA (Con) transfected cells (*, *p* < 0.05; **, *p* < 0.01; ***, *p* < 0.001) or between different transfections as indicated (#, *p* < 0.05). ANOVA, analysis of variance; VHL, von Hippel‐Lindau; HIF‐1α, hypoxia‐inducible factor 1α; PHD, prolyl hydroxylase domain; PDK1, pyruvate dehydrogenase kinase 1; PAI1, plasminogen activator inhibitor 1; Epo, erythropoietin; REPD, REP cell‐derived; RT‐qPCR, reverse transcription quantitative polymerase chain reaction

Pharmaceutical PHD inhibition is known to restore Epo expression in anemic ESRD patients whose REP cells appear to be in an “off” state. FG‐4592 (roxadustat) was the first PHD inhibitor approved for the treatment of renal anemia (Dhillon, 2019). Therefore, we exposed AB‐REPD2‐22 cells to nonpermissive (37°C) conditions for 14 days, followed by treatment with FG‐4592. As shown in Figure 2a, FG‐4592 time‐dependently induced the transcript levels of the HIF target PHD2 (but not PHD3) to a similar extent as hypoxia, with variable induction factors between independent experimental series. In contrast, Epo mRNA mainly showed strong fluctuations without any clear induction by FG‐4592, indicating only sporadic transcriptional conversion into “on” cells.

As PHD2/3 and VHL deletions have been reported to result in (sometimes even ectopic) constitutive Epo expression (Gerl et al., 2015; Kobayashi et al., 2016; Minamishima et al., 2008; Takeda et al., 2008), we used RNA interference in AB‐REPD2‐22 cells to stably knock‐down PHD2, PHD3, and VHL, alone or in the indicated combinations. As shown in Figure 2b, knock‐down efficiency generally was >90%. Only PHD2 and PHD2/VHL silencing led to an increase in the normoxic and hypoxic expression of the HIF target PHD3. PHD3 silencing and, surprisingly, VHL silencing did not lead to an increase in the HIF target PHD2 (Figure 2b). Because we previously identified PHD2 as a HIF‐1 and PHD3 as a HIF‐1/2 target (Stiehl et al., 2012), these results suggest that HIF‐2 rather than HIF‐1 target genes respond to silencing of PHD‐dependent oxygen signaling in REPD cells cultured under nonpermissive conditions.

HIF‐1α mRNA remained unaffected by these knock‐downs or hypoxia, and the hypoxic induction of the HIF‐1 target PDK1 (pyruvate dehydrogenase kinase 1) was not altered by PHD2, PHD3, or VHL single knock‐downs, and even attenuated by the PHD2/PHD3 and PHD3/VHL double‐knock‐downs (Figure 2c, upper panel). In contrast, HIF‐2α mRNA was strongly induced by PHD2 silencing (but not hypoxia) in REPD cells, additive to the nonpermissive conditions. PHD3 and VHL knock‐down had no significant effect on HIF‐2α mRNA, and PHD2/PHD3 but not PHD2/VHL double‐knock‐down attenuated the effect of PHD2 silencing (Figure 2c, lower panel). The HIF‐2‐specific target plasminogen activator inhibitor 1 (PAI1) was induced by PHD2 and VHL but not PHD3 silencing. Transcript levels of the HIF‐2 target Epo in the same samples were only induced upon PHD2 silencing, once more to a quite variable degree in the independent experiments. (Co‐)silencing of VHL had no effect and PHD3 cosilencing even abolished the PHD2 knock‐down effect in these cells (Figure 2c, lower panel).

In summary, while suppression of cellular proliferation and PHD2 silencing could specifically enhance HIF‐2α and HIF‐2‐dependent PAI1 and Epo gene expression, presumably by oxygen‐independent mechanisms, this procedure did not convert REPD cells to permanently “on” Epo expressing cells compared with the established hepatoma and neuroblastoma cell culture models. In line with this conclusion, while HIF‐1α protein levels remained high we could not detect HIF‐2α protein by immunoblotting, despite the increase in HIF‐2α mRNA levels by SV40 large T inactivation and PHD2 silencing (Figures S2A, B).

### 3.3 Increased number of mitochondria‐rich TNTs in hypoxia and role of mitochondrial localization for oxygen‐sensing in REPD cells

**Figure 3:**
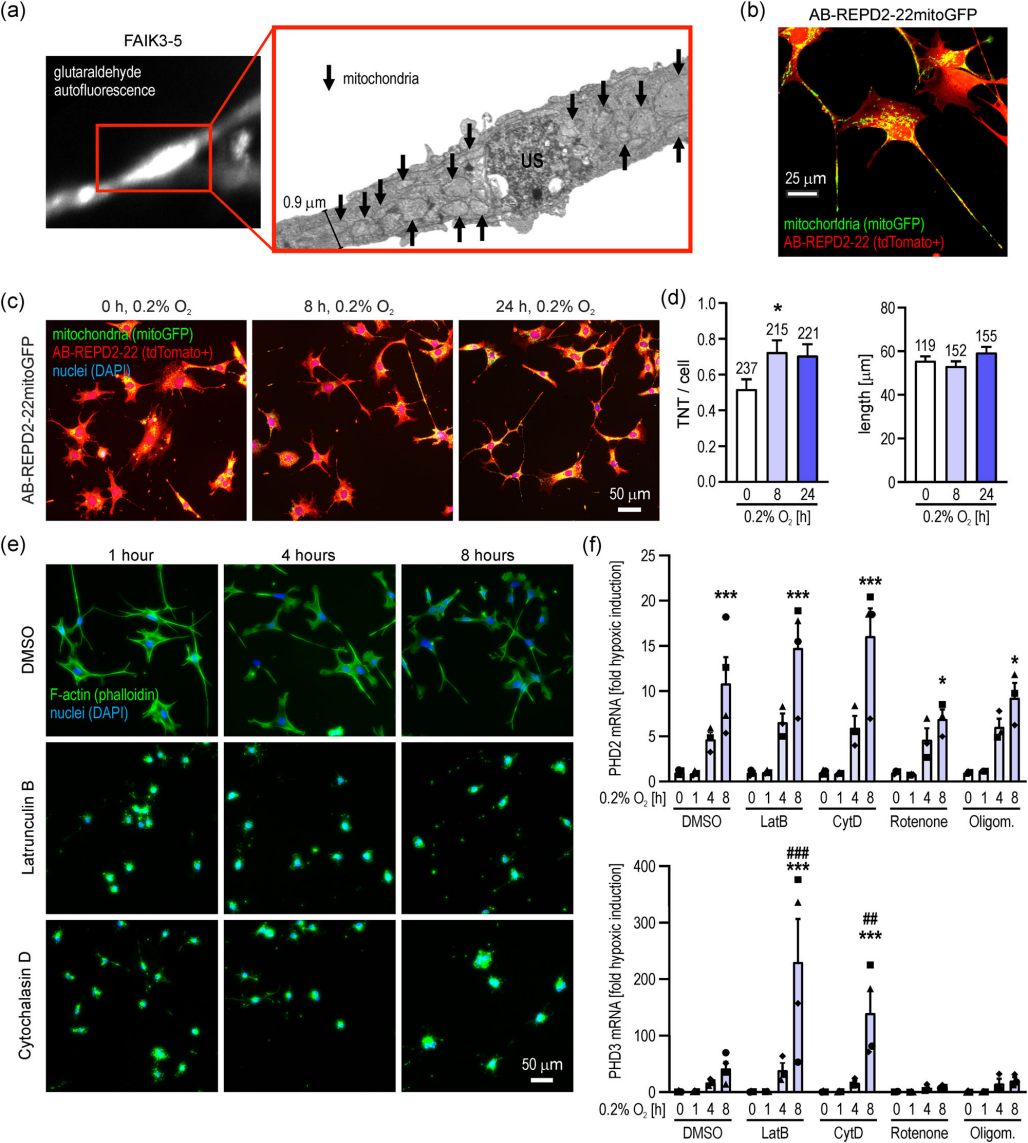
Mitochondria and tunneling nanotube (TNT) function in REPD cells. (a) Fluorescence microscopy of a contact area between two TNTs of FAIK3‐5 REPD cells, visualized by glutaraldehyde autofluorescence (white; left panel) and focussed ion beam scanning electron microscopy of the same area (right panel). Mitochondria and an unidentified electron‐dense structure at the contact point are indicated by arrows and "US", respectively. (b) Maximal *z*‐projection fluorescence microscopy of tdTomato+ AB‐REPD2‐22mitoGFP cells (yellow color indicates tdTomato/mitoGFP double‐positive pixels). (c) Fluorescence microscopy of AB‐REPD2‐22mitoGFP cells kept under nonpermissive (37°C) conditions for 14 days followed by exposure to hypoxia as indicated. (d) Quantification of TNT number (left panel) and length (right panel) of the AB‐REPD2‐22 cells shown in (c). The number of analyzed cells and TNTs is indicated. Shown are mean values + SEM. One‐way ANOVA with Dunnett's multiple comparisons test was used to evaluate the difference to the respective normoxic control (*, *p* < 0.05). (e) Fluorescence microscopy of AB‐REPD2‐22 cells treated with the actin polymerization inhibitors latrunculin B or cytochalasin D following cultivation under nonpermissive conditions (37°C) for 14 days. F‐actin and nuclei were stained with phalloidin (green) and DAPI (blue), respectively. (f) Hypoxic induction of the HIF targets PHD2 and PHD3 in AB‐REPD2‐22 cells treated with solvent (DMSO), actin polymerization inhibitors (LatB or CytD) or mitochondrial complex I (Rotenone) or V (Oligom.) inhibitors following cultivation under nonpermissive conditions (37°C) for 14 days. mRNA levels were determined by RT‐qPCR, normalized to the ribosomal protein L28 mRNA levels, and shown relative to the normoxic controls (mean +  SEM, *n* = 3 to 4). Two‐way ANOVA with Dunnett's multiple comparisons tests were used to evaluate the effects of hypoxia versus normoxia (*, *p* < 0.05; ***, *p* < 0.001) and inhibitors versus solvent (^##^, *p* < 0.01; ^###^, *p* < 0.001). ANOVA, analysis of variance; FAIK, fibroblastoid atypical interstitial kidney; DAPI, 4', 6‐diamidino‐2‐phenylindole; HIF, hypoxia‐inducible factor; PHD, prolyl hydroxylase domain; REPD, REP cell‐derived; RT‐qPCR, reverse transcription quantitative polymerase chain reaction

We have previously shown that mitochondria are present in the protrusions of REP cells in vivo as well as of FAIK cell lines in vitro (Imeri et al., 2019). Mitochondria within these long protrusions represent a typical feature of the so‐called TNTs (Vignais et al., 2017). Interestingly, focussed ion beam scanning electron microscopy of a TNT from FAIK3‐5 cells showed that not only do the TNT bulges contain mitochondria but mitochondria are also densely packed in the thinner parts of the TNTs (Figure 3a). Of note, a suspected contact site of two TNTs in the center of the bulge showed an unidentified electron‐dense structure. To analyze mitochondrial shape and distribution further, AB‐REPD2‐22 cells were stably transfected with mitochondria green fluorescent protein (mitoGFP) (Figure 3b). Following cultivation under nonpermissive (37°C) conditions for 14 days, these cells were exposed to hypoxia for up to 24 h (Figure 3c), which led to a significant induction of the number of TNTs per cell, while the average length remained unchanged (Figure 3d).

To investigate a potential role of the mitochondria‐rich TNTs in oxygen sensing, AB‐REPD2‐22 cells were kept under nonpermissive (37°C) conditions for 14 days and treated with the actin polymerization inhibitors latrunculin B and cytochalasin D. As shown in Figure 3e, even after 1 h of treatment TNTs were largely absent and the cells acquired a round morphology. Despite these profound morphological changes, oxygen‐sensing was not impaired as demonstrated by the unaltered hypoxic induction of the HIF target PHD2 and even a significant further induction of PHD3 after 8 h of hypoxic exposure (Figure 3f). In contrast, inhibition of the mitochondrial electron transport chain complexes I and V with rotenone and oligomycin, respectively, reduced hypoxic PHD2 induction and mostly abolished PHD3 induction (Figure 3f). These results indirectly suggest that only central but not peripheral mitochondrial oxygen consumption influences normal oxygen sensing in REPD cells in vitro.

### 3.4 Pericyte but not chondrogenic, osteogenic, or adipogenic differentiation of REPD cells in vitro

**Figure 4:**
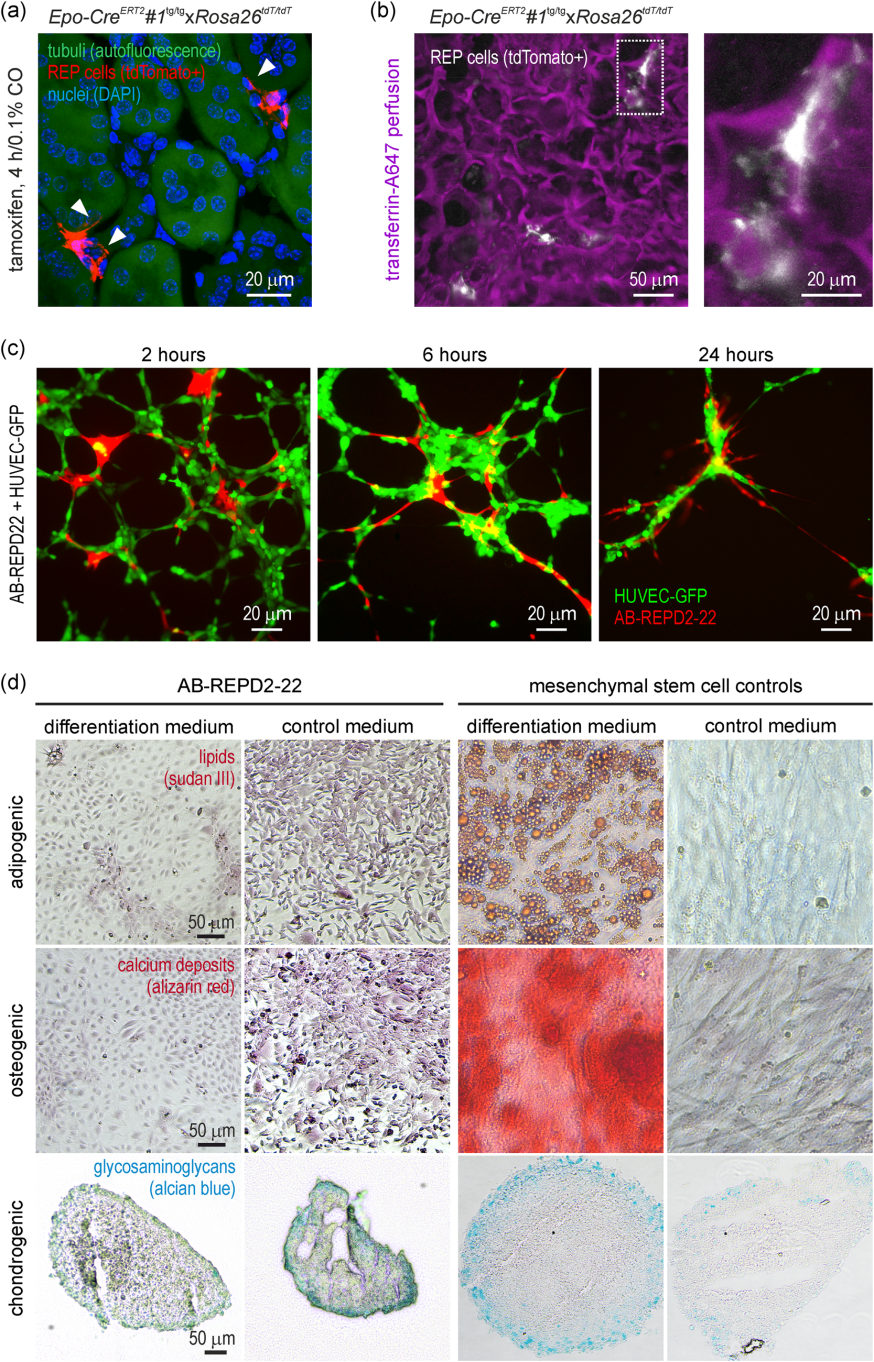
Pericyte but not MSC‐like features of REPD cells. (a) Fluorescence microscopy of tdTomato+ REP cells (red) in vivo. The REP cell protrusions extending into the interstitial space along tubular structures are indicated by arrowheads. Tubuli were visualized by their autofluorescence (green) and nuclei by DAPI (blue). (b) Intravital multiphoton imaging of the association of tdTomato+ REP cells (white) with vessels perfused with labeled transferrin (magenta). (c) Fluorescence microscopy of tdTomato+ AB‐REPD2‐22 cells (red) cultivated under nonpermissive conditions (37°C) for 14 days followed by coculture with GFP‐labeled HUVECs (green). (d) Bright‐field images of fixed AB‐REPD2‐22 cells (stained as indicated) after cultivation under nonpermissive (37°C) conditions for 7 days followed by the indicated differentiation media for 14 (osteogenic) or 21 (adipogenic, chondrogenic) days. Adipose tissue‐derived MSCs served as positive controls. REP, renal Epo‐producing; REPD, REP cell‐derived; GFP, green fluorescent protein; DAPI, 4', 6‐diamidino‐2‐phenylindole; MSC, mesenchymal stem cell; HUVEC, human umbilical vein endothelial cell

As shown in Figure 4a, the TNTs of REP cells in vivo extend into the intertubular space. As REP cells are known to express typical markers of pericytes, including PDGFRβ, neuron‐glial antigen 2 (NG2), and forkhead box D1 (FOXD1) (Asada et al., 2011; Chang et al., 2016; Gerl et al., 2016; Imeri et al., 2019; Kobayashi et al., 2016), it is likely that these TNTs primarily align with peritubular blood vessels, as previously quantified in a mouse model with constitutively labeled REP cells (Souma et al., 2016). We confirmed these findings by intravital tdTomato+ REP cell imaging of mouse kidneys following perfusion with fluorescently labeled transferrin, demonstrating that more TNTs appeared to associate with perfused blood vessels than with (nonlabeled) tubules (Figure 4b).

To investigate whether REPD cells adopt a pericyte‐like phenotype in vitro, including the interaction with vascular endothelial cells, human umbilical vein endothelial cells were stably transfected with GFP (HUVEC‐GFP) and cultured in matrigel to form a vessel‐like network. Soon after the addition of tdTomato+ AB‐REPD2‐22 cells, preincubated under nonpermissive (37°C) conditions for 14 days, long TNT protrusions were formed which readily aligned with the “tube walls” generated by the HUVEC‐GFP cells (Figure 4c). Virtually all AB‐REPD2‐22 cells were in contact with HUVEC‐GFP cells within the first 2 h and extended their protrusions along the vascular structures during the next 4 h. REPD cells under permissive conditions, as well as nonconditionally immortalized REPD cells, showed a similar association with endothelial cells (data not shown), suggesting that the pericyte‐like behavior is a robust feature of this cell type.

As pericyte differentiation and/or REP‐endothelial cell–cell contacts belong to the possible microenvironmental factors required for Epo expression, we exposed HUVEC‐REPD cocultures to hypoxic conditions and analyzed Epo mRNA levels. However, we did not find any relevant change in Epo expression under these culture conditions (data not shown).

Regarding their fibroblast‐like nature, REPD cells would have been expected to de‐differentiate into MSC‐like cells under nonpermissive conditions. To analyze their MSC‐like differentiation potential, we cultured AB‐REPD2‐22 cells for 7 days under nonpermissive (37°C) conditions and replaced the cultivation medium by media designed for adipogenic, osteogenic, or chondrogenic differentiation, or kept the cells in an MSC culture medium as control. However, in contrast to MSC controls, none of these media resulted in phenotypic changes characteristic for MSC‐like cells (Figure 4d).

### 3.5 Neurogenic differentiation of conditionally immortalized REPD cells in vitro

Transgenically labeled REP cells in the adult mouse kidney have been shown to coexpress the neuronal markers microtubule‐associated protein 2 (MAP2) and neurofilament light polypeptide (NFL) (Obara et al., 2008). Furthermore, it has been suggested that the majority of REP cells originates from myelin protein zero lineage‐labeled extrarenal cells entering the embryonic kidney at E13.5 and that attenuated Epo production in the diseased kidney could be restored by the administration of neurotrophins (Asada et al., 2011).

**Figure 5:**
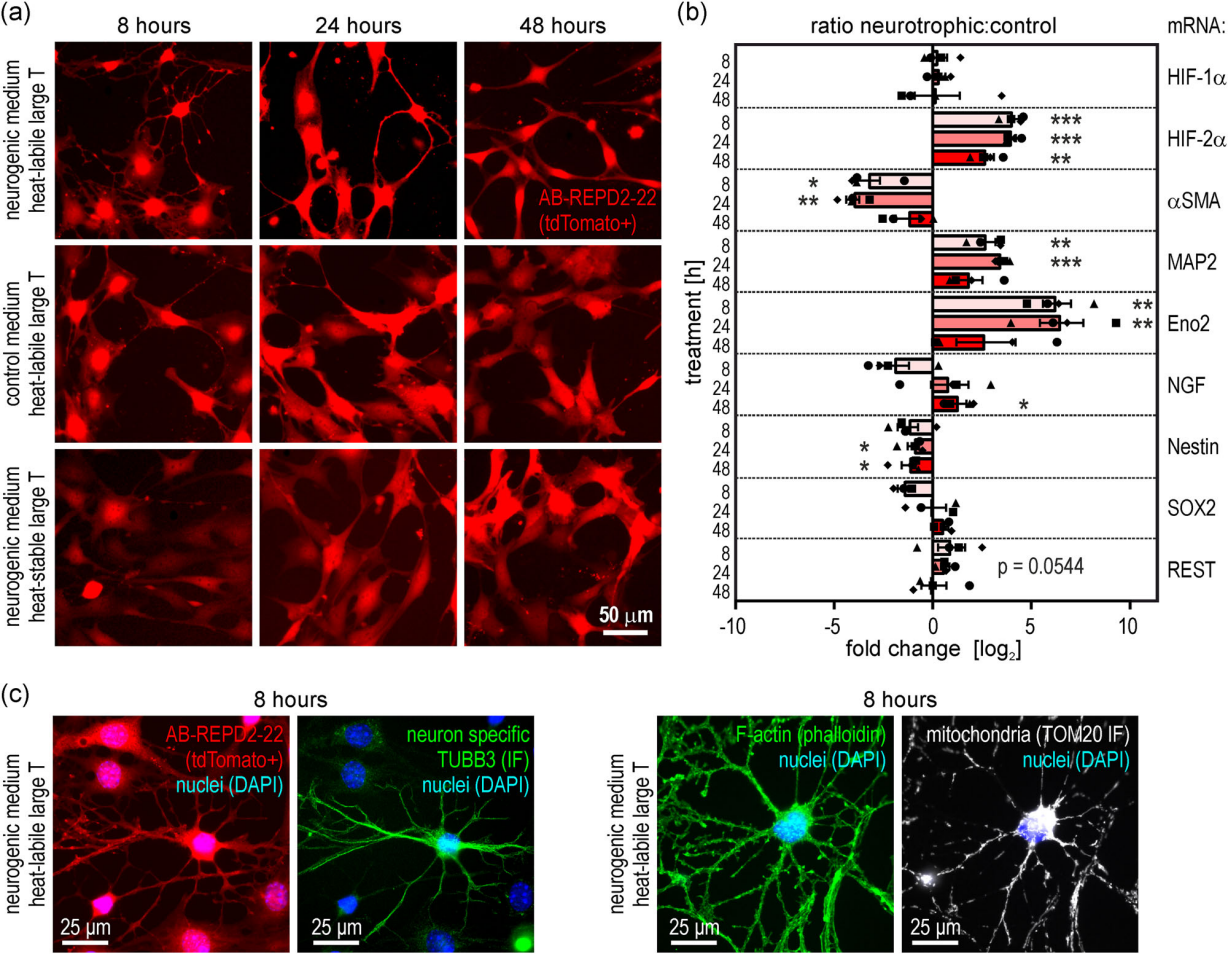
Neurogenic differentiation of REPD cells. (a) Fluorescence microscopy of tdTomato+ AB‐REPD2‐22 cells (red) incubated under nonpermissive conditions (37°C) for 7 days followed by incubation in a neurotrophic differentiation medium (top panel) or a control medium (middle panel) for the indicated time periods. AB‐REPD2‐22 cells additionally transfected with a wild‐type large T antigen served as a supplementary control (bottom panel). (b) Transcript changes in AB‐REPD2‐22 cells cultured under nonpermissive (37°C) conditions in neurotrophic compared with control media for the indicated time periods. mRNA levels were determined by RT‐qPCR, divided by the respective β‐actin mRNA levels, and shown as log_2_ of the fold change (mean ±* SEM*, *n* = 4). The delta‐delta *C*_t_ method ($2^{‐\Delta \Delta C_{t}}$) was used where no internal standard was available. Ratio paired *t* tests were used to evaluate differences between neurotrophic and control medium (*, *p* < 0.05; **, *p* < 0.01; ***, *p* < 0.001). HIF, hypoxia‐inducible factor; αSMA, α smooth muscle actin; MAP2, microtubule‐associated protein 2; Eno2, enolase 2; NGF, nerve growth factor; SOX2, Sry‐related high‐mobility box 2; REST, RE1‐silencing transcription factor/neuron‐restrictive silencer factor (NRSF). (c) Expression of neuron‐specific TUBB3 (tubulin α3) and mitochondrial density (TOM20 immunofluorescence; IF) in outgrowths of AB‐REPD2‐22 cells cultured as in (b). REPD, REP‐derived; RT‐qPCR, reverse transcription quantitative polymerase chain reaction

To analyze the neurogenic differentiation potential of REPD cells in vitro, AB‐REPD2‐22 cells were cultured for 7 days under nonpermissive (37°C) conditions followed by transfer into a neurotrophic differentiation medium or a control medium. As an additional control, AB‐REPD2‐22 were stably “super‐transfected” with wild‐type SV40 large T antigen to allow for proliferative cultivation at 37°C. After 8 h in the neurotrophic medium, most cells formed numerous thin processes reminiscent of astrocytes and neurons. With ongoing culture, these cells acquired a highly elongated shape, reminiscent of radial glial neuronal progenitor cells. In contrast, AB‐REPD2‐22 cells additionally containing the heat‐stable SV40 large T antigen did not show any neurogenic differentiation (Figure 5a).

In addition to the induction by nonpermissive conditions, the expression of HIF‐2α (but not HIF‐1α) mRNA was further induced upon neurotrophic stimulation (Figure 5b), but the HIF‐2α protein still remained noninduced (Figure S2C). Although the mRNA levels of the myofibroblast marker αSMA were reduced, the neuronal markers MAP2 and Eno2 (enolase 2) were strongly induced (Figure 5b). The changes in the glial markers were less conclusive, with significantly elevated nerve growth factor (NGF) levels only after 48 h and downregulated nestin levels. Also, the transcription factors Sry‐related high‐mobility box 2 (SOX2) and RE1‐silencing transcription factor/neuron‐restrictive silencer factor (REST), indicating stem cell and neuronal properties, respectively, did not show any conclusive changes in the same samples (Figure 5b).

Neurogenic differentiation was further supported by increased expression of neuron‐specific TUBB3 (tubulin β3), known to play an essential role in axon outgrowth (Qu et al., 2013). TUBB3 was particularly located in the outgrowths of those cells that adopted an early neuron‐like phenotype (Figure 5c). Mitochondria were still present in these outgrowths, albeit at apparently lower levels (Figure 5c). Overall, these data suggest that AB‐REPD2‐22 cells cultivated in cellular quiescence have neurogenic properties and differentiate into a neuron‐like state upon neurotrophic stimulation.

## 4 DISCUSSION

Whereas REP cells in vivo normally do not proliferate (Dahl et al., 2022; Souma et al., 2013), immortalized REPD cells in vitro divide approximately twice a day (Imeri et al., 2019), limiting the application as a REP cell model. Therefore, we conditionally immortalized freshly isolated “on” REP cells using a heat‐labile SV40 large T antigen (Bapst et al., 2020). As shown herein, following cultivation under nonpermissive (37°C or 39°C) conditions, these REPD cells ceased to proliferate and strongly induced the expression of HIF‐2α but not HIF‐1α as well as the stem cell markers SCA‐1 and CD133. HIF‐2 is not only the master regulator of hypoxic Epo expression but has also been reported to induce octamer‐binding transcription factor 4 (OCT4), Nanog homeobox (NANOG), and sex determining region Y‐box 2 (SOX2), the three main transcription factors involved in embryonic stem cell maintenance (Petruzzelli et al., 2014), altogether suggesting cellular de‐differentiation under large T antigen inhibitory conditions.

Despite these enormous phenotypic changes, REPD cells under nonpermissive conditions did not recapitulate the permanent hypoxia‐inducible Epo regulation known from the “classic” hepatoma and neuroblastoma cell culture models (Goldberg et al., 1987; Stolze et al., 2002). Neither pharmaceutic nor genetic procedures to (permanently) stabilize HIFα improved Epo induction. However, expectations of a REPD cell culture model are mainly driven by the vast experience with the use of these cancer cell models derived from nonrenal tissue. There are various mechanistic differences in Epo regulation between REP cells of the kidney and hepatocytes of the liver, or neurons, astrocytes, and pericytes of the central nervous system (Bernaudin et al., 2000; Imeri et al., 2019; Marti et al., 1996; Orlando et al., 2020; Semenza et al., 1991). REP cells in vivo display a transient “on‐off” behavior, even under ongoing hypoxic conditions, with apparently stochastic recruitment from a large pool of usually silent but potentially Epo‐production‐capable cells (Dahl et al., 2022; Souma et al., 2013, 2015; Suzuki & Yamamoto, 2016). Therefore, a true REP cell culture model probably should recapitulate this poorly understood stochastic “on‐off” process, rather than behave like hepatic and neuronal malignant cell lines.

PHD2 has previously been reported to be the main HIFα modifying enzyme and PHD2 knock‐out mice show an enormous increase in Epo mRNA in the liver (Minamishima et al., 2008; Takeda et al., 2008). The role of PHD3 is much less understood. Intriguingly, in REPD cells cosilencing of PHD3 abolished the HIF‐2α and Epo‐inducing effect of PHD2 silencing. A similar phenomenon has recently been reported in VHL‐deficient clear cell renal cell carcinoma where PHD3 silencing downregulated HIF‐2α mRNA stability (Miikkulainen et al., 2019; Zacharias et al., 2021). Thus, PHD3 might specifically regulate yet unidentified factors that directly or indirectly affect HIF‐2α and/or Epo expression. Such factors could include epigenetic modifications as previously reported for the mouse *Epo* locus (Chang et al., 2016; Sato, Kumagai, et al., 2019). PHD3 has also been shown to hydroxylate pyruvate kinase M2 (PKM2) which enhances the binding of PKM2 to HIF‐1α and promotes the transactivation of HIF‐1 target genes (Luo et al., 2011; Zheng et al., 2021). However, it is currently unknown whether a similar mechanism also applies to HIF‐2α.

Cultured REPD cells, especially under nonpermissive conditions, recapitulated many morphological and phenotypic features of telocyte‐like REP cells in vivo. We found a close and rapid association with endothelial cells and the long processes readily aligned with the vascular structures. These processes of cultured REPD cells contain F‐actin, α‐tubulin, and densely packed mitochondria, hallmarks of TNTs (Vignais et al., 2017). Coincident with the loss of Epo expression and myofibroblast transdifferentiation, TNTs of REP cells have been shown to realign from vascular to tubular structures during kidney disease (Souma et al., 2016), suggesting a role of TNTs in Epo regulation. It would be an attractive hypothesis that TNTs of pericytic REP cells “sense” the oxygen gradient that forms by hemoglobin oxygen desaturation along the longitudinally aligned peritubular blood capillaries. Oxygen sensing by TNTs may involve the oxygen‐consuming function of the highly abundant local mitochondria. During kidney disease, ultimately resulting in interstitial fibrosis, the TNT–vessel interaction appears to be disrupted (Souma et al., 2016), which may destroy the differential oxygen signal. Our in vitro experiments demonstrated that the number (but not length) of TNTs per REPD cell increased under hypoxic conditions, which could have been interpreted as an enlargement of the oxygen‐sensing capacity under chronically hypoxic conditions. However, at least under the cell culture conditions used, we provided evidence that only oxygen consumption by mitochondria of the cell body but not the TNTs (including their local mitochondria) is required for normal oxygen‐signaling in REPD cells. Intriguingly, the hypoxic induction of the HIF target gene PHD3 (but not PHD2) was significantly induced by the removal of TNTs, a finding that deserves further investigation.

As CD133^+^/CD73^+^ mesenchymal progenitor cells isolated from the inner medulla of the kidney have been reported to express Epo (Bussolati et al., 2013), we expected that REPD cells under nonpermissive conditions would adopt MSC‐like features, including the potential to differentiate into the adipogenic, osteogenic, and chondrogenic lineages. Although nonpermissive conditions led to a strong increase of the stem cell markers SCA‐1 and CD133, following stimulation with the appropriate media REPD cells did not differentiate into these lineages. Interestingly, REPD cells rather differentiated into the neurogenic lineage upon cultivation in a neurotrophic medium. Following rapid morphological and transcriptional changes toward astrocyte/neuron‐like cells, REPD cells converted to radial glia‐like cells after prolonged cultivation in the neurotrophic medium, further demonstrating the high plasticity of cultured REPD cells.

Despite the additive induction of HIF‐2α mRNA by nonpermissive combined with neurogenic cell culture conditions, HIF‐2α protein remained undetectable. This may be explained by simultaneous myofibroblastic transforming growth fator α (TGF‐α) signaling under nonpermissive conditions, indicated by the αSMA increase, which has recently been shown to inhibit the HIF‐2α‐Epo axis (Fuchs et al., 2021; Shih et al., 2021). Although the simultaneous increases in stem cell and myofibroblast markers appear contradictory, it should be noted that seven distinct cell clusters with increasing αSMA levels can be distinguished during myofibroblast differentiation (Ó hAinmhire et al., 2019). Low‐level αSMA induction may hence reflect TGF‐β signaling in REP cells occurring before full myofibroblast transdifferentiation. Moreover, the remaining high HIF‐1α protein levels may antagonize HIF‐2α, a phenomenon that has been well characterized in cancer cell lines (Stiehl et al., 2012).

In summary, the conditionally immortalized REPD cells presented in this study recapitulate phenotypic features of REP cells in vivo and serve as a useful tool for the in vitro analyses of this enigmatically plastic cell type. We found pronounced neuronal features of these cells, in line with the observation of neuronal markers in vivo.

## CONFLICT OF INTERESTS

The authors declare that there are no conflict of interests.

## AUTHOR CONTRIBUTIONS

Andreas M. Bapst, Thomas Knöpfel, Karen A. Nolan, Faik Imeri, Claus D. Schuh, and Jia Guo performed experiments. Andreas M. Bapst analyzed the results. Andrew M. Hall and Dörthe M. Katschinski provided technical and intellectual input. Roland H. Wenger designed the research and wrote the article. All authors approved the final manuscript.

## Supporting information

Supporting information.

## Data Availability

Underlying raw data can be obtained from Roland H. Wenger (roland.wenger@access.uzh.ch) and are available via https://dataverse.harvard.edu/ using the accession code https://doi.org/10.7910/DVN/OSCG6Z.
