# Supplementary material for: Neurogenic and pericytic plasticity of conditionally immortalized cells derived from renal erythropoietin‐producing cells
Source: J Cell Physiol. 2022 Jan 10;237(5):2420–33. doi: 10.1002/jcp.30677 (PMC9303970; doi:10.1002/jcp.30677)
Supplement: Supplementary file 1 — Supporting information. [file JCP-237-2420-s001.pdf]

SUPPORTING INFORMATION

Supplementary Table S1

Antibodies used for immunofluorescence

**Antigen**	**Type**	**Product Number**	**Company**
NFL	chicken polyclonal	ab72997	Abcam, Cambridge, UK
CD133	rabbit polyclonal	NB120-16518	Novus Biologicals, Littleton, USA
PDGFRβ	rabbit monoclonal	ab32570	Abcam, Cambridge, UK
MAP2	rabbit polyclonal	sc-20172	Santa Cruz Biotechnology, Dallas, USA
CD73	rat monoclonal	BD TY/23	BD Biosciences, Allschwil, Switzerland
TOM20	rabbit polyclonal	sc-17764	Santa Cruz Biotechnology, Dallas, USA
TUBB3	mouse monoclonal	TUJ1	Biolegend, San Diego, USA
SCA-1	rat monoclonal	ab51317	Abcam, Cambridge, UK
Secondary goat antibodies coupled to Alexa 488 or 647			Thermo Fisher Scientific, Waltham, USA.

Antibodies used for immunoblotting

**Antigen**	**Type**	**Product Number**	**Company**
HIF-1α	rabbit polyclonal	NB100-449	Novus Biologicals, Littleton, USA
HIF-2α	rabbit polyclonal	PAB12124	Abnova, Taipeh, Taiwan
HIF-2α	rabbit polyclonal	A-700-002-T	Bethyl, Montgomery, TX, USA
α-tubulin	rabbit polyclonal	2144	Cell Signaling, Danvers, MA, USA
Tbp	mouse monoclonal	ab818	Abcam, Cambridge, UK
Secondary goat anti-rabbit (HRP)		31460	Thermo Fisher Scientific, Waltham, USA.
Secondary goat anti-mouse (HRP)		31430	Thermo Fisher Scientific, Waltham, USA.

Supplementary Table S2

RT-qPCR primers

Acta2_fwd	5’-gactactgccgagcgtgag-3’
Acta2_rev	5’-gtcagcaatgcctgggtaca-3’
Actb_fwd	5’-ccagccttccttcttgggtat-3’
Actb_rev	5’-cttctgcatcctgtcagcaa-3’
Eno2_fwd	5’-agccctcatcagctcaggta-3’
Eno2_rev	5’-ctgagcaatgtggcgataga-3’
Epo_fwd	5’-aatggaggtggaagaacagg-3’
Epo_rev	5’-acccgaagcagtgaagtga-3’
Hif1a_fwd	5’-acacagaaatggcccagtga-3’
Hif1a_rev	5’-ttcacaaatcagcaccaagc-3’
Hif2a_fwd	5’-ggacgctctgcctatgagtt-3’
Hif2a_rev	5’-cagccacacatatcctccgt-3’
L28_fwd	5'-gcaaaggggtcgtggtagtt-3'
L28_rev	5'-ttctggcttcgaaggatggc-3'
Map2_fwd	5’-gccagcctcagaacaaacag-3’
Map2_rev	5’-aaggtcttgggagggaagaac-3’
Nestin_fwd	5’-ctgcaggccactgaaaagtt-3’
Nestin_rev	5’-aggtgtctgcaagcgagagt-3’
Ngf_fwd	5’-gcagtgaggtgcatagcgta-3’
Ngf_rev	5’-ctgtgtcaagggaatgctga-3’
Pai1_fwd	5’-ccaacatcttggatgctgaa-3’
Pai1_rev	5’-ctgctcttggtcggaaagact-3’
Pdk1_fwd	5’-ggcggctttgtgatttgtat-3’
Pdk1_rev	5’-acctgaatcgggggataaac-3’
Phd2_fwd	5’-gcaacggaacaggctatgtc-3’
Phd2_rev	5’-ctcgctcatctgcatcaaaa-3’
Phd3_fwd	5’-caacttcctcctgtccctca-3’
Phd3_rev	5’-ggctggacttcatgtggatt-3’
Rest_fwd	5’-gtgcgaactcacacaggaga-3’
Rest_rev	5’-aagaggtttaggcccgttgt-3’
Sca1_fwd	5’-tcttgtggccctactgtgtg-3’
Sca1_rev	5’-ggcagatgggtaagcaaaga-3’
Sox2_fwd	5’-aagggttcttgctgggtttt-3’
Sox2_rev	5’-agaccacgaaaacggtcttg-3’
Vhl_fwd	5’-atccacagctaccgaggtca-3’
Vhl_rev	5’-cttccgcacacttgggtagt-3’

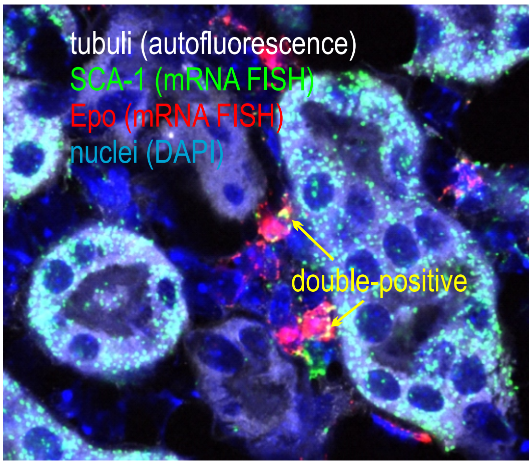

Supplementary Figure S1. SCA-1 expression in REP cells *in vivo*. mRNA fluorescence *in situ* hybridization (mRNA-FISH) of Epo (red) and SCA-1 (green) in a kidney derived from a mouse exposed for 4 hours to 0.1% CO. Tubuli were visualized by their autofluorescence (white) and nuclei were stained with DAPI (blue). Yellow arrows indicate SCA-1 positive REP cells.

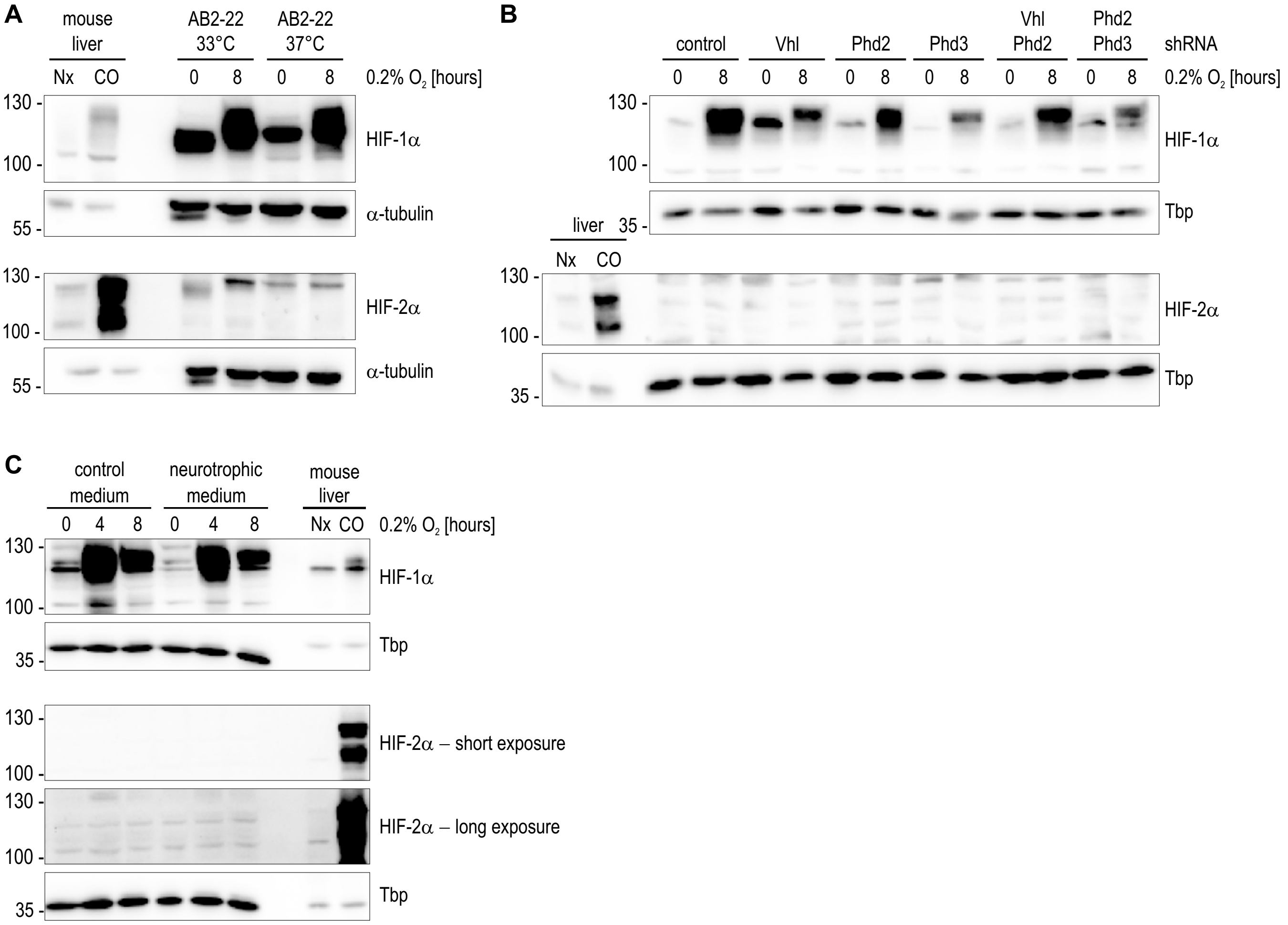

Supplementary Figure S2. HIFα protein levels in REPD cells. Immunoblotting of HIF-1α and HIF-2α using extracts of REPD cells cultured under conditions that have been found to specifically induce HIF -2α mRNA levels. Where indicated, HIFα induction in extracts of livers derived from mice exposed to 0.1% carbon monoxide for 4 hours (CO) vs. normoxic control mice (Nx) was used as positive control. (**A**) AB2-22 REPD cells were exposed to permissive (33°C) or non-permissive (37°C) conditions for 14 days, followed by exposure to 0.2% for 8 hours. α-Tubulin served as loading/blotting control. (**B**) AB2-22 REPD cells with shRNA-mediated knockdown of the indicated genes were exposed as above. Nuclei were isolated and nuclear extracts were immunoblotted . TATA-box-binding protein (Tbp) served as loading/blotting control. (**C**) AB2-22 REPD cells were cultured under control or neurotrophic conditions followed by treatment, extraction and immunoblotting as in **B**. Short and long exposure times are shown for the HIF-2α immunoblot, indicating treatment-independent faint normoxic background HIF-2α protein levels.
